# *Small Ubiquitin-Like Modifier 4 (SUMO4)* Gene M55V Polymorphism and Type 2 Diabetes Mellitus: A Meta-analysis Including 6,823 Subjects

**DOI:** 10.3389/fendo.2017.00303

**Published:** 2017-11-02

**Authors:** Yan-yan Li, Hui Wang, Xin-xing Yang, Hong-yu Geng, Ge Gong, Hyun Jun Kim, Yan-hong Zhou, Jing-jing Wu

**Affiliations:** ^1^Department of Gerontology, First Affiliated Hospital of Nanjing Medical University, Nanjing, China; ^2^Institute of Clinical Medicine, First Affiliated Hospital of Nanjing Medical University, Nanjing, China; ^3^Department of Cardiology, First Affiliated Hospital of Nanjing Medical University, Nanjing, China; ^4^Department of Gerontology, Nanjing General Hospital, Nanjing, China; ^5^Department of Physiology, University of Cincinnati, Cincinnati, OH, United States; ^6^Department of Nephrology, First Affiliated Hospital of Nanjing Medical University, Nanjing, China

**Keywords:** *small ubiquitin-like modifier 4*, rs237025, polymorphism, type 2 diabetes mellitus, meta-analysis

## Abstract

**Background:**

Many studies suggest that the *small ubiquitin-like modifier 4 (SUMO4)* M55V gene polymorphism (rs237025) may be associated with an increased risk of type 2 diabetes mellitus (T2DM). However, due to other conflicting results, a clear consensus is lacking in the matter.

**Objective and methods:**

A meta-analysis consisting of 6,823 subjects from 10 studies was conducted to elucidate relationship between the *SUMO4* M55V gene polymorphism and T2DM. Depending on the heterogeneity of the data, either a fixed or random-effects model would be used to assess the combined odds ratio (ORs) and their corresponding 95% confidence interval (CI).

**Results:**

*SUMO4* gene M55V polymorphism was significantly associated with T2DM in the whole population under allelic (OR: 1.18, 95% CI: 1.10–1.28, *P* = 1.63 × 10^−5^), recessive (OR: 1.59, 95% CI: 1.14–2.23, *P* = 0.006), dominant (OR: 0.815, 95% CI: 0.737–0.901, *P* = 6.89 × 10^−5^), homozygous (OR: 1.415, 95% CI: 1.170–1.710, *P* = 0.0003), heterozygous (OR: 1.191, 95% CI: 1.072–1.323, *P* = 0.001), and additive genetic models (OR: 1.184, 95% CI: 1.097–1.279, *P* = 1.63 × 10^−5^). In our subgroup analysis, a significant association was found again in the Chinese population, but not in Japanese or Iranian population.

**Conclusion:**

*SUMO4* gene M55V polymorphism may correlate with increased T2DM risk. Chinese carriers of the V allele of the *SUMO4* gene M55V polymorphism may be predisposed to developing T2DM.

## Introduction

Type 2 diabetes mellitus (T2DM) is a long-term metabolic disorder characterized by high blood sugar, insulin resistance, and impaired insulin secretion. Recently, research found the nuclear factor-κB (NF-κB) signaling pathway to be mechanistically implicated in the pathogenesis of T2DM by increasing pancreatic β-cell apoptosis (1–3). Small ubiquitin-like modifier 4 (SUMO4), a newly discovered molecule located in insulin-dependent diabetes mellitus 5 (IDDM5), has been shown to suppress the transcription of NF-κB. SUMO4 also acts as an anti-oxidant, protecting the pancreatic β-cells from oxidative damage and promoting β-cell survival (4). Therefore, under oxidative stress by autoimmune processes, SUMO4 and the DNA damage signaling protein iKB can be mobilized to activate intracellular pathways associated with cell survival (5).

The *SUMO4* gene is located at position 6q25 in the IDDM5 locus and spans 688 bp. The gene contains a single exon, encoding 95 amino acids (6). The M55V polymorphism is caused by mutation of an adenosine at the 1633d position to a guanine (rs237025, A163G). The conformational change caused by the polymorphism results in increased NF-κB transcriptional activity and, thus, increased expression of NF-κB-dependent genes.

Though researchers have found good evidence for an association between this polymorphism and T2DM susceptibility, the exact relationship is still debated, possibly due to the varying genetic backgrounds of different ethnic groups (7). In this regard, other associations with T2DM, such *HMGA1* and *TCFTL2* variants, have been found to be heterogeneous across different ethnic groups (8, 9).

In 2004, Bohren et al. found the *SUMO4* Met55Val gene polymorphism to be associated with an increased susceptibility to type 1 diabetes mellitus (T1DM) in the Caucasian population (6). Around the same time, Guo et al. replicated the result in a Chinese population (4). In 2005, Noso et al. found Val 55 to be significantly more common in T1DM patients than in control subjects in the Japanese population (4). In 2008, Ikegami et al. has performed the genome-wide association (GWA) study and found that the M55V variant was significantly associated with T1DM in the Asian populations, but not Caucasian populations (10). Other studies have suggested the susceptibility loci for T2DM may lie in the area on Chromosome 6 that *SUMO4* is located (11–14), indicating a common genetic basis for both types of diabetes. Considering the possible functional evidence for *SUMO4* as a candidate gene for T2DM, Noso et al. initially explored the contribution of the *SUMO4* Met55Val locus to T2DM susceptibility and found a significant association in a Japanese population (15). Ji et al. found similar results among a Chinese Han population in the Hubei region. Compared with control MM genotype, Ji et al. found that individuals with the VV or MV genotype to have higher levels of fasting insulin and Homeostasis Model Assessment-Insulin Resistance (7). A study by Pu et al. also came upon the same result in a Beijing population in China (16). However, the results on the matter are not unanimous: in 2010, Fallah et al. found that *SUMO4* gene M55V variant was not associated with the susceptibility of T2DM in an Iranian population (17).

We conducted the current meta-analysis from 3,223 T2DM patients and 3,600 controls to verify the association of *SUMO4* M55V gene polymorphism and T2DM.

## Materials and Methods

### Publication Search and Inclusion Criteria

The included studies should meet the following inclusion criteria: (a) assessment of the association of *SUMO4* gene M55V polymorphism with T2DM; (b) T2DM diagnosis and classification meets guidelines proposed by the World Health Organization in 1999: fasting blood sugar no less than 7.0 mmol/l and the 2 h postprandial blood sugar no less than 11.1 mmol/l. Other metabolic conditions, such as acute and chronic complications of diabetes mellitus, T1DM, ketosis, hepatic and renal dysfunction, and other conditions were excluded. (c) Officially published case–control or cohort studies. (d) Genotype in control group follows Hardy–Weinberg equilibrium (HWE).

The following electronic databases were used to conduct a search: China National Knowledge Infrastructure, VIP database, Wanfang database, China Biological Medicine Database, and PubMed. Keywords used for the search were “SUMO4, rs237025, diabetes.” When searching Chinese databases, researchers employed corresponding Chinese terms. Nine publications were retrieved in our initial search of the Pubmed database and four papers met the inclusion criteria for this meta-analysis. Using keyword combination “SUMO4, Met55Val, diabetes” yielded three additional papers, all of which were eligible for our analysis. The eligible studies’ characteristics conform to the above inclusion criteria. Finally, “SUMO4, M55V, type 2 diabetes” retrieved another two additional papers. Both papers met inclusion criteria. Additional three Chinese papers were retrieved in the China National Knowledge Infrastructure database by using the keywords combination as “*small ubiquitin-like modifier 4*, Met55Val, diabetes.” Retrieved studies were published between 2003 and 2017.

### Data Extraction

Data were extracted by three investigators using a standardized protocol (Table 1). Two investigators were responsible for identifying duplicate studies while the third acted as the mediator to resolve any disagreements between them. Studies that deviated from the major inclusion criteria were published in duplicate, or provided insufficient data were rejected. Similar data sets published in different articles by a single author group were adopted once in the meta-analysis. Items, such as the first author’s name, publication year, ethnicity, matching criteria, genotype number, and total number of cases and controls, are displayed in Table 1.

**Table 1:** Characteristics of the investigated studies of the association between *small ubiquitin-like modifier 4 (SUMO4)* gene M55V polymorphism and T2DM.

Reference	Year	Ethnicity	T2DM			Control			Matching criteria	Genotype method	Sample size (T2DM/control)
			MM	MV	VV	MM	MV	VV			
Pu et al. (16)	2012	Chinese	160	218	26	250	218	32	Age, sex, ethnicity	PCR-HRM	404/500
Li et al. (22)	2011	Chinese	110	94	28	44	51	7	Ethnicity	PCR-RFLP	232/102
Shimada et al. (23)	2009	Japanese	395	393	86	412	414	79	Ethnicity	TaqMan SNP genotyping assay	874/905
Lin et al. (24)	2010	Chinese	275	254	45	186	115	22	Ethnicity	PCR-RFLP	574/323
Noso et al. (15)	2007	Japanese	146	166	43	200	158	40	Ethnicity	TaqMan SNP genotyping assay	355/398
Fallah et al. (17)	2010	Iranian	10	22	18	13	25	12	Age, sex, ethnicity	PCR-RFLP	50/50
Ji et al. (7)	2010	Chinese	195	185	46	143	123	15	Age, sex, ethnicity	PCR-RFLP	427/281
Hu and Song (25)	2009	Chinese-Va	59	31	6	68	33	3	BMI, ALT, Cr, UA, TC, TG, HDL-C, LDL-C, ethnicity	PCR-RFLP	96/104
Hu and Song (25)	2009	Chinese-Lalu	40	11	3	168	60	6	Age, sex, ethnicity, BMI, WHR, SBP, DBP, HDL-C, Hypertension percentage	PCR-RFLP	54/234
Wang et al. (26)	2015	Chinese	45	54	1	249	218	32	Age, BMI, DBP, TG, LDL-C, ethnicity	PCR-RFLP	100/499
Zhang et al. (27)	2017	Chinese	25	22	11	105	90	9	Sex, BMI, Hypertension percentage, Hyperlipidemia percentage	PCR-RFLP	58/204

*T2DM, type 2 diabetes mellitus; TC, total cholesterol; LDL, low-density lipoprotein-cholesterol; PCR-RFLP, polymerase chain reaction-restriction fragment length polymorphism; PCR-HRM, polymerase chain reaction-high resolution melting curve*.

*PCR-RFLP and case–control study design were adopted in the above studies*.

### Statistical Analysis

Statistical analyses were performed by using Revman 5.0 and STATA 12.0 software (StataCorp, College Station, TX, USA). Six genetic models, allelic (V allele distribution frequency), recessive (VV vs. MV + MM), dominant (MM vs. MV + VV), homozygous (VV vs. MM), heterozygous (MV vs. MM), and additive (total V vs. total M), were used in the current meta-analysis. The association of *SUMO4* gene M55V polymorphism and T2DM were compared by using the odds ratios (ORs) corresponding to its 95% confidence intervals (CIs).

The heterogeneity between the individual studies was evaluated by using the Chi-square-based Q-test with significance set at *P* < 0.05 level (18). If heterogeneity was detected in the studies, the random-effects model (DerSimonian and Laird method) would be used to estimate the pooled OR (19). If not, the fixed-effects model (the Mantel–Haenszel method) would be used (20). The combined OR was determined by *Z*-test with the significance set at *P* < 0.05 level.

Fisher’s exact test was used to assess HWE with the significance set at *P* < 0.05 level. The potential publication bias was evaluated by the funnel plot. The funnel plot symmetry was assessed by using the Egger’s linear regression test on the OR and the significance was set at *P* < 0.05 level (21).

## Results

### Studies and Populations

After the retrieval process, 10 papers met the inclusion criteria. Data were extracted from a total of 3,223 T2DM patients and 3,600 controls (Table 1) (7, 15–17, 22–27). Among the retrieved studies, one study was excluded for controls that deviated from the HWE (28). Three ethnicities were represented in the meta-analysis: Chinese, Japanese, and Iranian.

### Pooled Analyses

**Figure 1:**
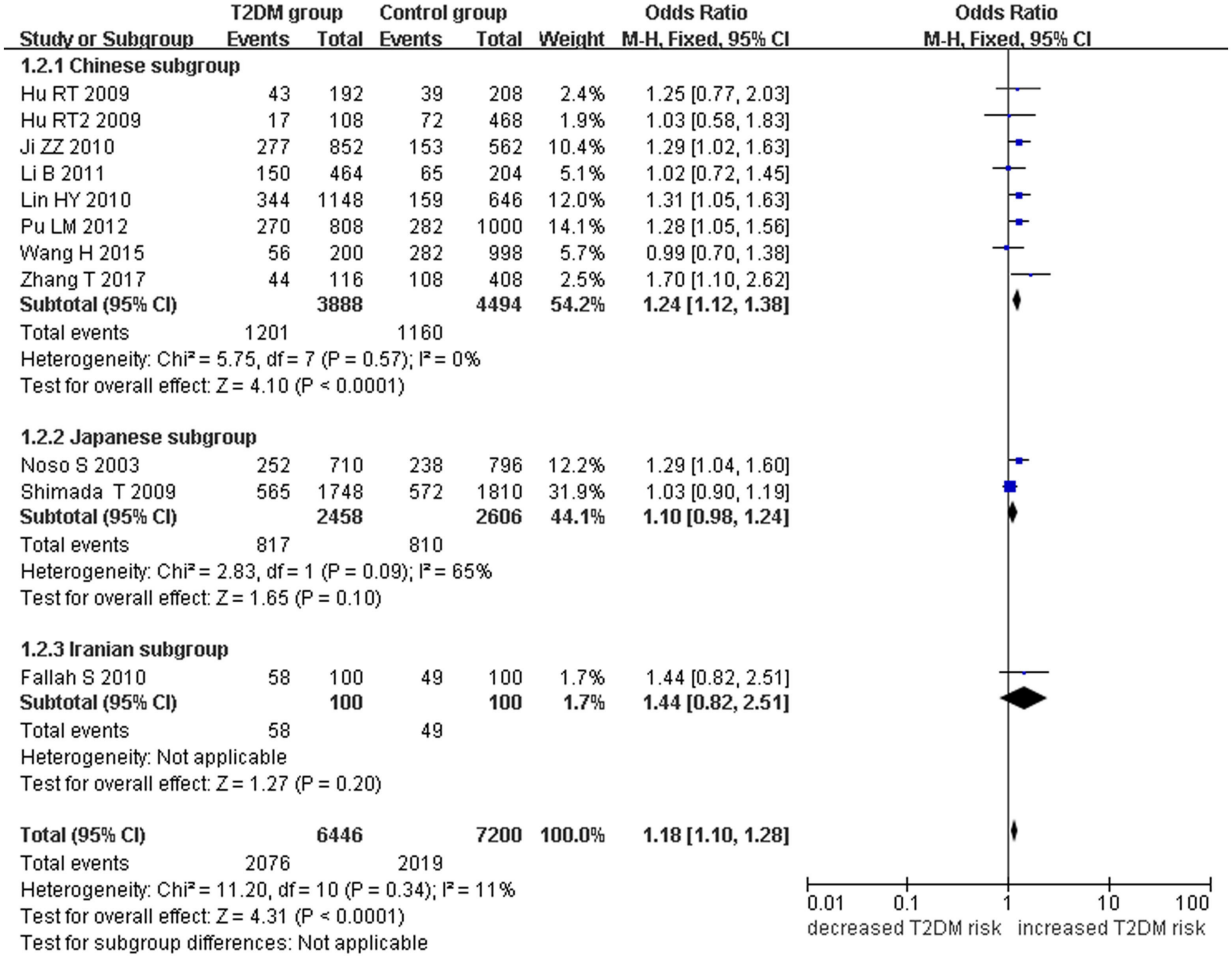
Forest plot of type 2 diabetes mellitus (T2DM) associated with *small ubiquitin-like modifier 4* (*SUMO4*) gene M55V polymorphism under an allelic genetic model (distribution of V allelic frequency of *SUMO4* gene).

**Figure 2:**
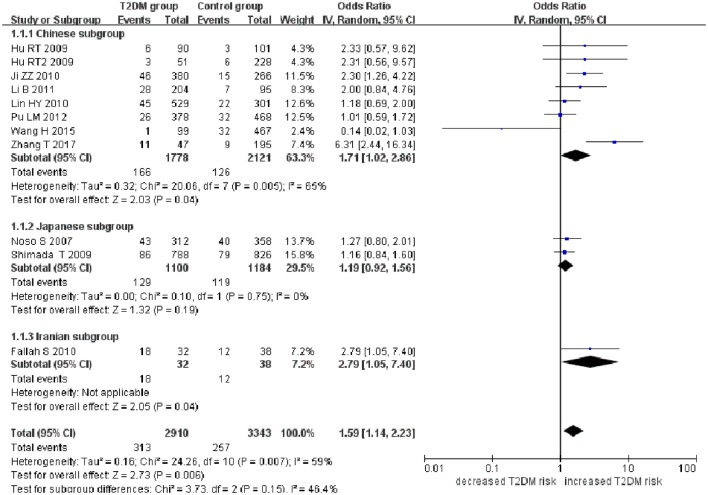
Forest plot of type 2 diabetes mellitus (T2DM) associated with *small ubiquitin-like modifier 4* gene M55V polymorphism under a recessive genetic model (VV vs. MM + MV).

**Figure 3:**
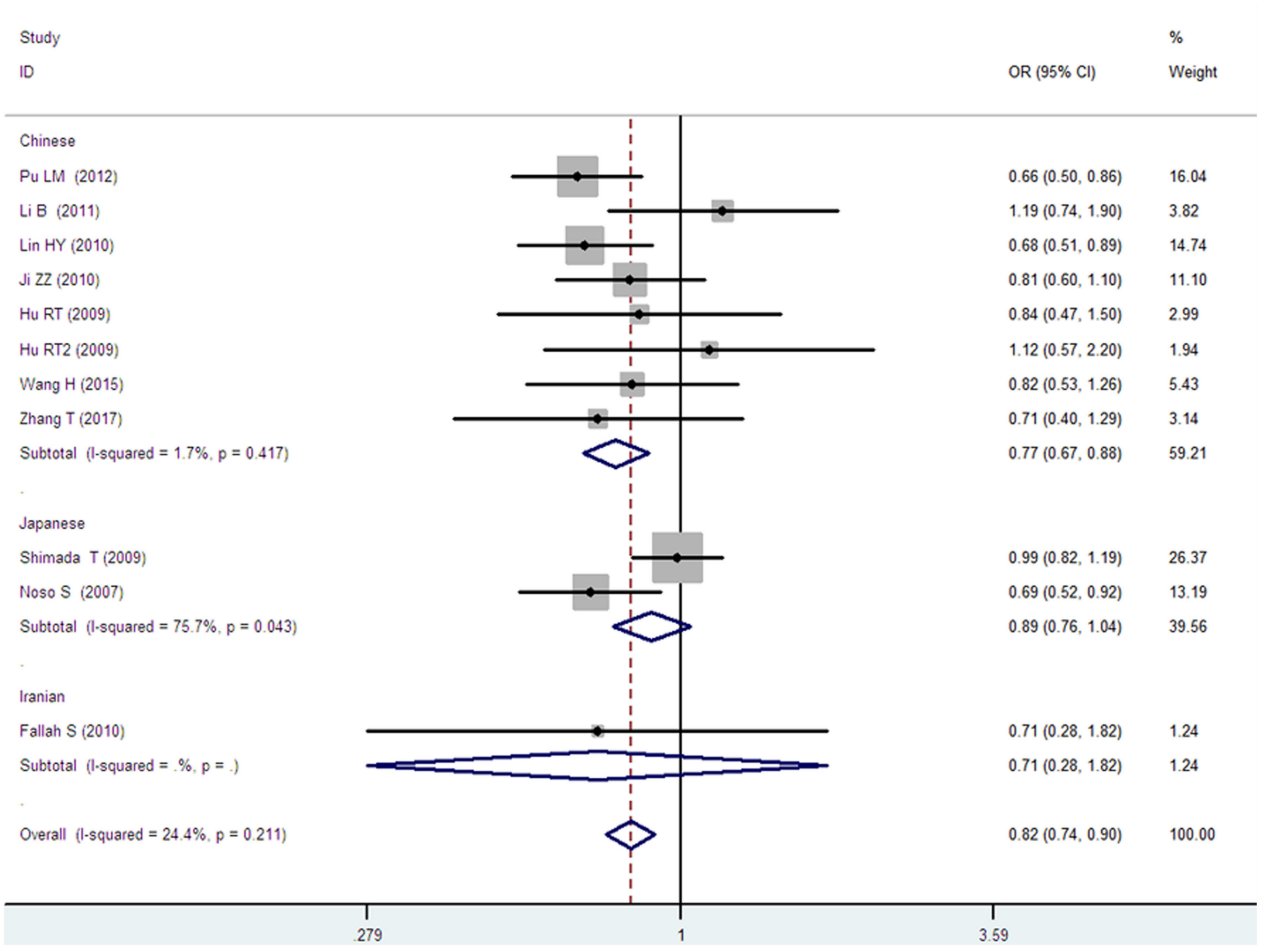
Forest plot of type 2 diabetes mellitus associated with *small ubiquitin-like modifier 4* gene M55V polymorphism under a dominant genetic model (MM vs. MV + VV).

**Figure 4:**
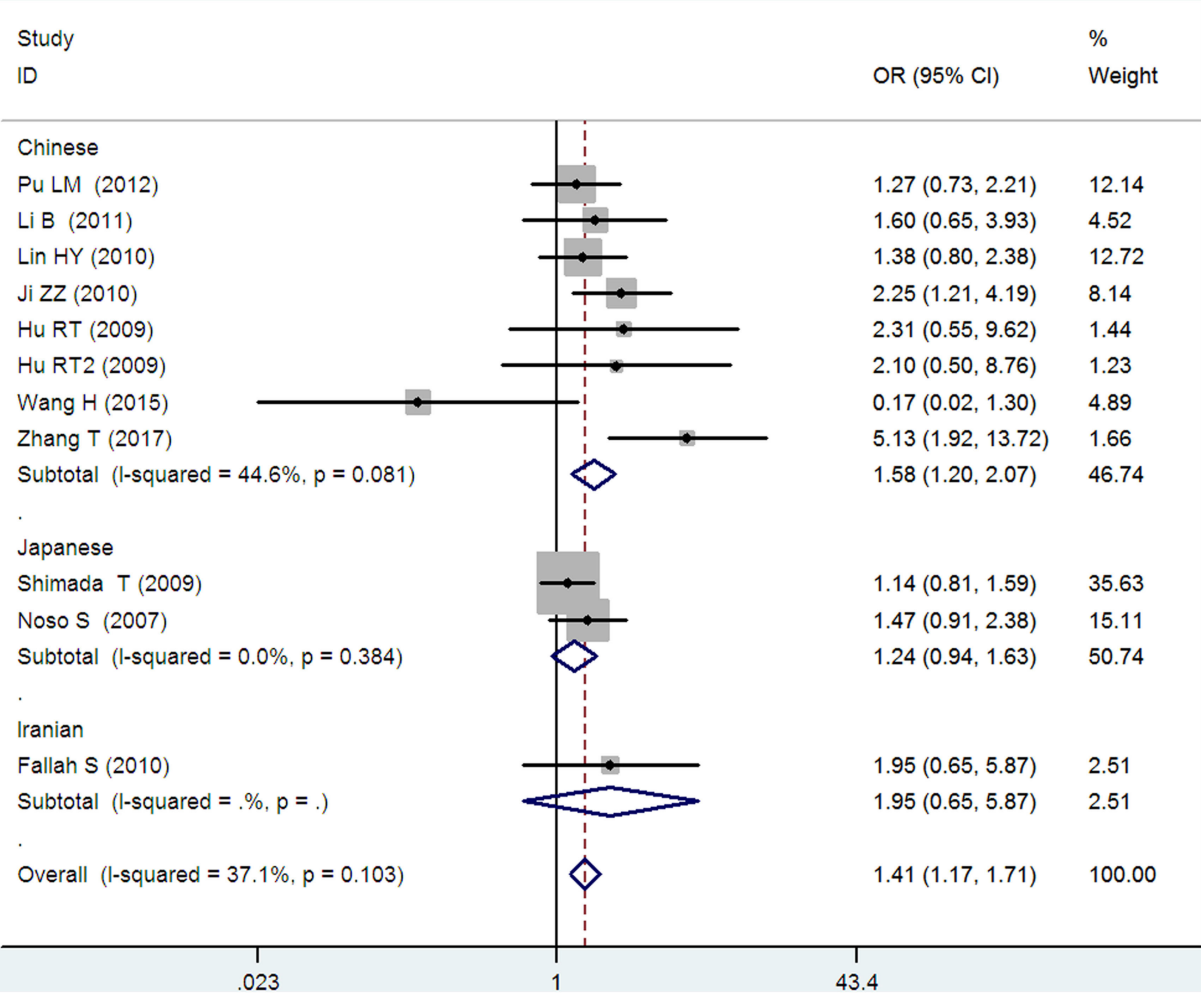
Forest plot of type 2 diabetes mellitus associated with *small ubiquitin-like modifier 4* gene M55V polymorphism under a homozygous genetic model (VV vs. MM).

**Figure 5:**
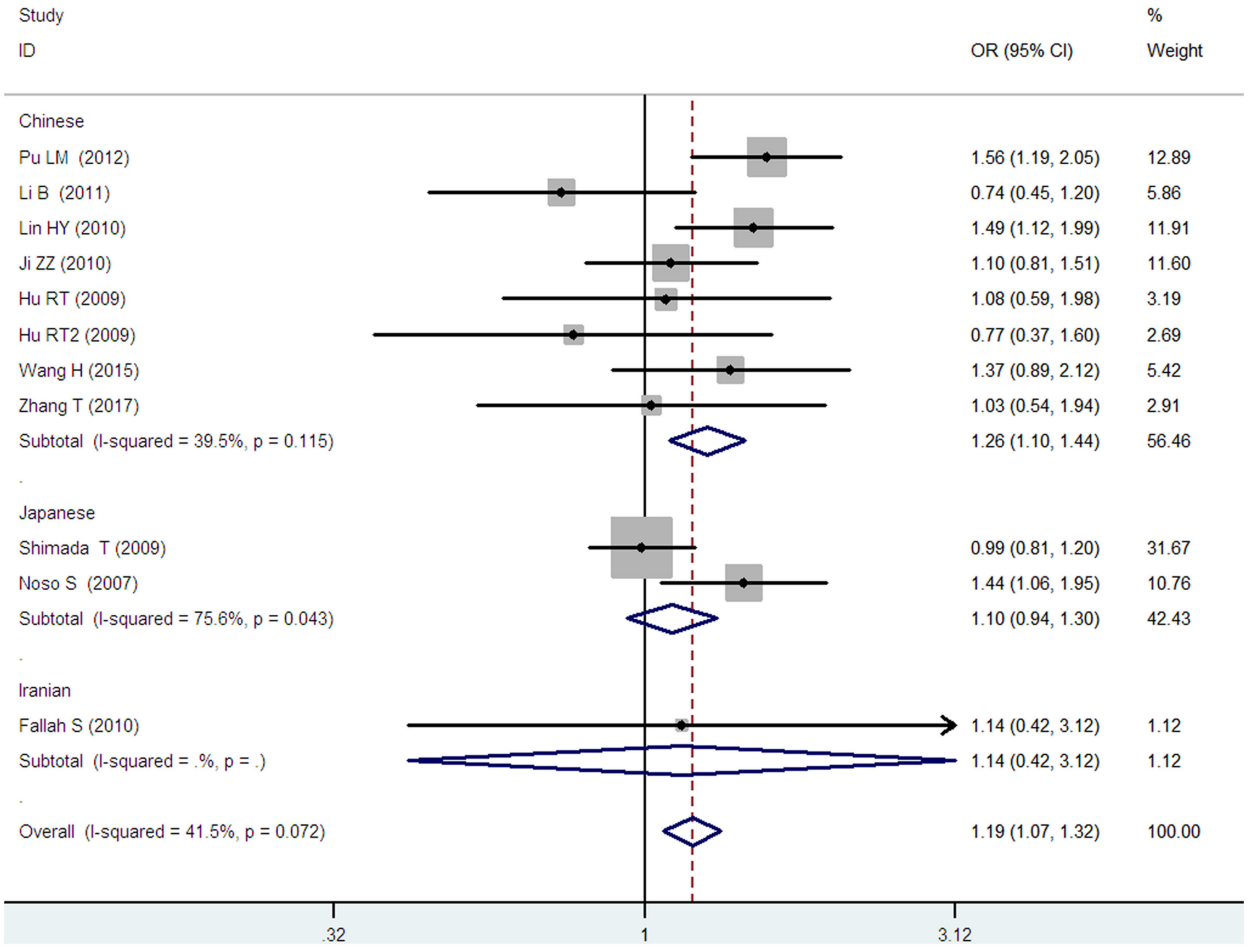
Forest plot of type 2 diabetes mellitus associated with *small ubiquitin-like modifier 4* gene M55V polymorphism under a heterozygous genetic model (MV vs. MM).

**Figure 6:**
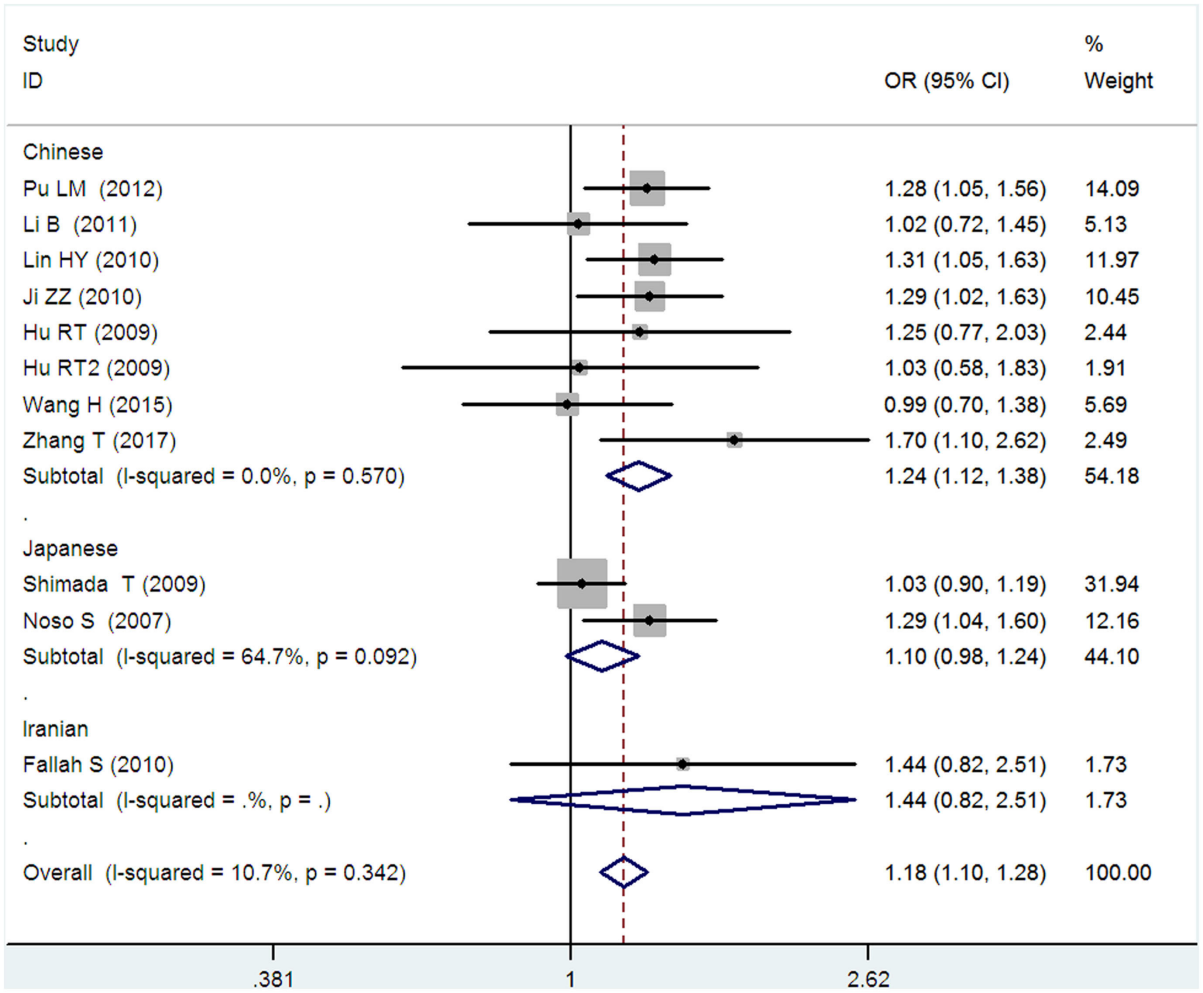
Forest plot of type 2 diabetes mellitus associated with *small ubiquitin-like modifier 4* gene M55V polymorphism under an additive genetic model (V vs. M).

*Small ubiquitin-like modifier 4* gene M55V polymorphism demonstrated a significant association with T2DM susceptibility in the whole population under allelic (OR: 1.18, 95% CI: 1.10–1.28, *P* = 1.63 × 10^−5^), recessive (OR: 1.59, 95% CI: 1.14–2.23, *P* = 0.006), dominant (OR: 0.815, 95% CI: 0.737–0.901, *P* = 6.89 × 10^−5^), homozygous (OR: 1.415, 95% CI: 1.170–1.710, *P* = 0.0003), heterozygous (OR: 1.191, 95% CI: 1.072–1.323, *P* = 0.001), and additive genetic models (OR: 1.184, 95% CI: 1.097–1.279, *P* = 1.63 × 10^−5^) (Figures 1–6; Table 2).

**Table 2:** Summary of meta-analysis of association between *small ubiquitin-like modifier 4 (SUMO4)* gene M55V polymorphism and type 2 diabetes mellitus (T2DM).

Genetic model	Pooled OR (95% CI)	*Z* value	*P*-value	Study number	T2DM size	control size	$P_{\text{heterogeneity(}I^{2}\text{\%)}}$
Allelic genetic model	1.18 (1.10–1.28)	4.31	1.63 × 10^−5*^	10	3,223	3,600	0.34 (11.0%)
Chinese subgroup	1.24 (1.12–1.38)	4.10	4.13 × 10^−5*^	7	1,944	2,247	0.57 (0%)
Japanese subgroup	1.10 (0.98–1.24)	1.65	0.10	2	1,229	1,303	0.09 (65.0%)
Iranian subgroup	1.44 (0.82–2.51)	1.27	0.20	1	50	50	NA

Recessive genetic model	1.59 (1.14–2.23)	2.73	0.006*	10	3,223	3,600	0.007* (59.0%)
Chinese subgroup	1.71 (1.02–2.86)	2.03	0. 04*	7	1,944	2,247	0.005* (65.0%)
Japanese subgroup	1.19 (0.92–1.56)	1.32	0.19	2	1,229	1,303	0.75 (0%)
Iranian subgroup	2.79 (1.05–7.40)	2.05	0.04*	1	50	50	NA

Dominant genetic model	0.815 (0.737–0.901)	3.98	6.89 × 10^−5*^	10	3,223	3,600	0.211 (24.7%)
Chinese subgroup	0.768 (0.673–0.877)	3.89	^0.0001*^	7	1,944	2,247	0.417 (1.7%)
Japanese subgroup	0.888 (0.760–1.039)	1.48	0.138	2	1,229	1,303	0.043* (75.7%)
Iranian subgroup	0.712 (0.279–1.818)	0.71	0.477	1	50	50	NA

Homozygous genetic model	1.415 (1.170–1.710)	3.58	0.0003*	10	3,223	3,600	0.103 (37.1%)
Chinese subgroup	1.580 (1.205–2.071)	3.31	0.001*	7	1,944	2,247	0.081 (44.6%)
Japanese subgroup	1.236 (0.939–1.627)	1.51	0.131	2	1,229	1,303	0.384 (0%)
Iranian subgroup	1.950 (0.648–5.867)	1.19	0.235	1	50	50	NA

Heterozygous genetic model	1.191 (1.072–1.323)	3.26	0.001*	10	3,223	3,600	0.072 (41.5%)
Chinese subgroup	1.257 (1.095–1.443)	3.26	0.001*	7	1,944	2,247	0.031* (66.2%)
Japanese subgroup	1.104 (0.937–1.301)	1.18	0.237	2	1,229	1,303	0.043* (75.6%)
Iranian subgroup	1.144 (0.419–3.122)	0.26	0.793	1	50	50	NA

Additive genetic model	1.184 (1.097–1.279)	4.31	1.63 × 10^−5*^	10	3,223	3,600	0.342 (10.7%)
Chinese subgroup	1.241 (1.119–1.377)	4.10	4.13 × 10^−5*^	7	1,944	2,247	0.570 (0%)
Japanese subgroup	1.104 (0.981–1.243)	1.65	0.099	2	1,229	1,303	0.092 (64.7%)
Iranian subgroup	1.437 (0.823–2.511)	1.27	0.203	1	50	50	NA

*P ≤ 0.05.

*CI, confidence interval; OR, odds ratio; T2DM size, the total number of T2DM cases; control size, the total number of control group; allelic genetic model, V allele distribution frequency; recessive genetic model, VV vs. MM + MV; dominant genetic model, MM vs. MV + VV; homozygous genetic model, VV vs. MM; heterozygous genetic model, MV vs. MM; additive genetic model, V vs. M*.

In the subgroup analysis, a significant association was also found in the Chinese population under allelic (OR: 1.24, 95% CI: 1.12–1.38, *P* = 4.13 × 10^−5^), recessive (OR: 1.71, 95% CI: 1.02–2.86, *P* = 0.04), dominant (OR: 0.768, 95% CI: 0.673–0.877, *P* = 1.57 × 10^−4^), homozygous (OR: 1.580, 95% CI: 1.205–2.071, *P* = 0.001), heterozygous (OR: 1.257, 95% CI: 1.095–1.443, *P* = 0.001), and additive genetic models (OR: 1.241, 95% CI: 1.119–1.377, *P* = 4.13 × 10^−5^).

No significant association was detected in Japanese population under allelic (OR: 1.10, 95% CI: 0.98–1.24, *P* = 0.10), recessive (OR: 1.19, 95% CI: 0.92–1.56, *P* = 0.19), dominant (OR: 0.888, 95% CI: 0.760–1.039, *P* = 0.138), homozygous (OR: 1.236, 95% CI: 0.939–1.627, *P* = 0.131), heterozygous (OR: 1.104, 95% CI: 0.937–1.301, *P* = 0.237), or additive genetic models (OR: 1.104, 95% CI: 0.981–1.243, *P* = 0.099).

In the Iranian population, a marginally significant association was detected only under recessive genetic model (OR: 2.79, 95% CI: 1.05–7.40, *P* = 0.04). No significant association was detected in Iranian population under allelic (OR: 1.44, 95% CI: 0.82–2.51, *P* = 0.20), dominant (OR: 0.712, 95% CI: 0.279–1.818, *P* = 0.477), homozygous (OR: 1.950, 95% CI: 0.648–5.867, *P* = 0.235), heterozygous (OR: 1.144, 95% CI: 0.419–3.122, *P* = 0.793) or additive genetic models (OR: 1.437, 95% CI: 0.823–2.511, *P* = 0.203).

As no significant heterogeneity was detected under allelic, dominant, homozygous, heterozygous, or additive genetic models (*P*_heterogeneity_ > 0.05), a fixed-effect model was used to determine the pooled OR. While only under the recessive genetic model, significant heterogeneity was detected (*P*_heterogeneity_ < 0.05) and random-effect model was used (Table 2).

### Bias Diagnostics

**Figure 7:**
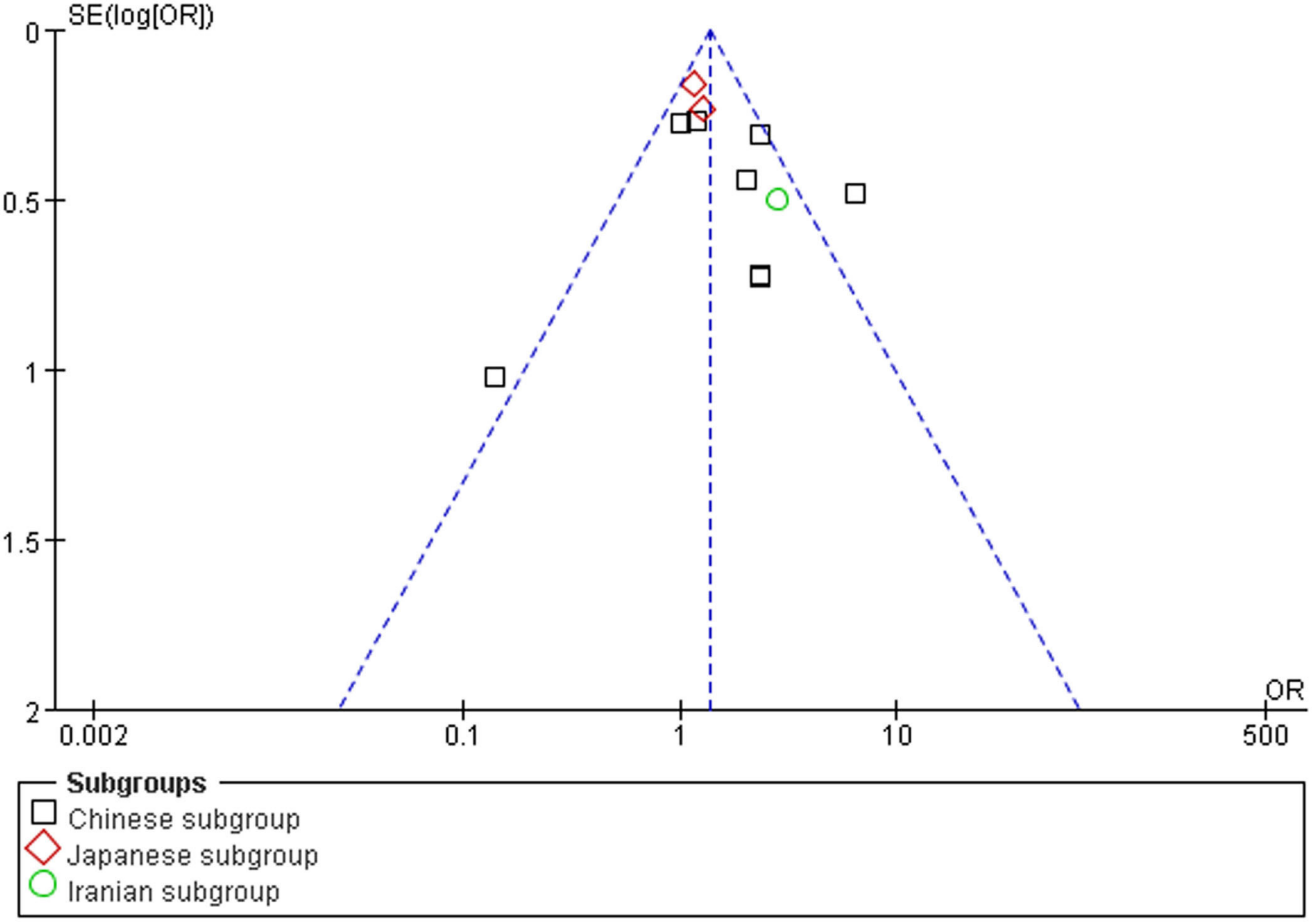
Funnel plot for studies of the association of type 2 diabetes mellitus and *small ubiquitin-like modifier 4* gene M55V polymorphism under a recessive genetic model (VV vs. MM + MV). The horizontal and vertical axis correspond to the OR and confidence limits. OR, odds ratio.

**Figure 8:**
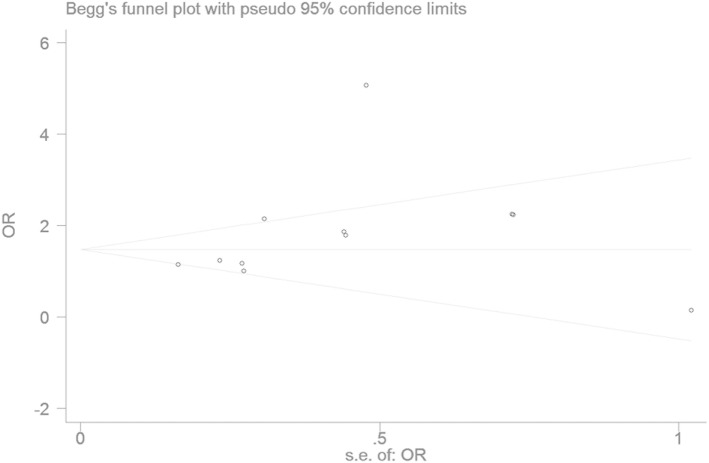
Begg’s funnel plot for studies of the association of type 2 diabetes mellitus and *small ubiquitin-like modifier 4* gene M55V polymorphism under a recessive genetic model (VV vs. MM + MV). The horizontal and vertical axis correspond to the OR and confidence limits. OR, odds ratio.

The publication bias of the individual studies was evaluated by using the funnel plot. No visual evidence for publication bias was evident in the funnel plot under the recessive genetic model (Figure 7). No significant publication bias was detected in this meta-analysis (*T* = 1.54, *P* = 0.159) under the recessive genetic model by using Egger’s test (Figure 8).

### Sensitivity Analysis

**Figure 9:**
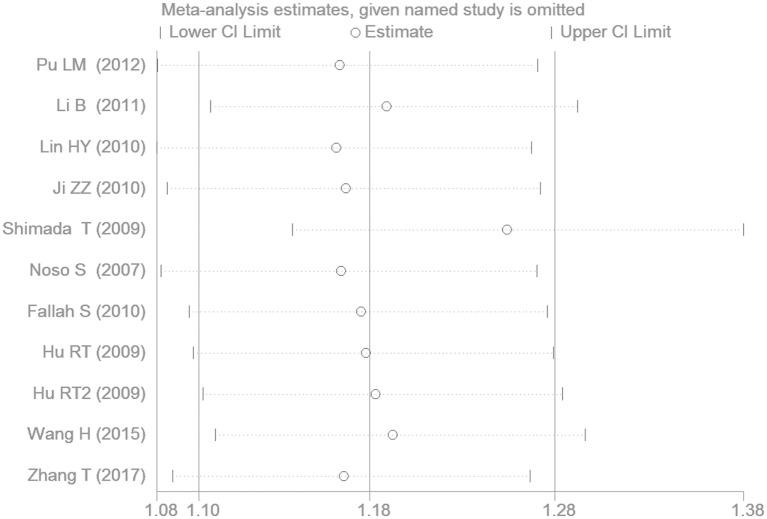
The sensitivity analysis results under the additive genetic model (V vs. M).

The removal of any one study from the meta-analysis did not change the significant association between *SUMO4* gene M55V polymorphism and T2DM under the additive genetic model, suggesting that the results are stable and robust (Figure 9).

## Discussion

In the current meta-analysis, a significant association was detected between *SUMO4* gene M55V polymorphism and T2DM under the allelic (OR: 1.18), recessive (OR: 1.59), dominant (OR: 0.815), homozygous (OR: 1.415), heterozygous (OR: 1.191), and additive (OR: 1.184) genetic models. In the subsequent subgroup analysis, a significant association was only detected in the Chinese population, but not in the Japanese or Iranian population. Significant heterogeneity was only found under the recessive genetic model. Heterogeneity was reduced in the Chinese and Japanese populations, suggesting that ethnicity was the main source for the heterogeneity. As only one study of the Iranian population was present in our analysis, the heterogeneity detection was not applicable.

The *SUMO4* gene M55V polymorphism is located in the evolutionarily crucial CUE structural domain and involves the substitution of the highly conserved Met (ATG) for Val (GTG). This substitution could modify site at which PKC phosphorylates the protein. The change in molecular conformation and functional activity reduces its ability to regulate NF-κB, resulting in increased NF-κB activation enhancement and overexpression of NF-κB-dependent gene products. In 2004, Guo et al. found that the M55V substitution resulted in 5.5 times greater NF-κB transcriptional activity and approximately two times greater expression of IL12B (4).

Nuclear factor-κB activation is an important molecule in the inflammatory response (29), upregulation of endothelin (30), and apoptosis (31). NF-κB activation may also promote apoptosis in both pericytes and endothelial cells in the pathogenesis of diabetes retinopathy (32).

In recent years, T2DM has been increasingly associated with mild, but systemic chronic inflammation that is characteristic of obesity and metabolic syndrome. The disruption of the internal equilibrium by factors, such as stress and poor nutrition, activates the inflammatory response as a protective response. Initially, the body is in a peri-inflammatory state, but long-term activation of this state can result in chronic inflammation (33). NF-κB is an important inflammatory factor involved in IR, but also acts as transcription factor for other pro-inflammatory proteins (34). The pivotal studies have also found that the SUMO4 cytosis substrate included the anti-oxidative stress proteins, the regulation proteins for DNA repair and synthesis, protein degradation associated proteins, glycometabolism-associated proteins under stress conditions induced by starvation (35). These findings point toward the functional complexity of SUMO4, which warrants further investigation and discussion.

In 2012, Tang et al. performed a meta-analysis on the relationship between *SUMO4* gene M55V polymorphism and T2DM where they found a significant association between them in the Asian population (*P* < 0.05) (36). However, only six individual studies were included and only four genetic models were adopted in their meta-analysis. In addition, with no subgroup analysis stratified by ethnicity, their conclusion may be more limited to the analysis we offer in this current study. In 2017, Zhang et al. performed a meta-analysis on this subject and came to a similar conclusion (37). However, only five Chinese individual studies published before 2012 were included in Zhang’s meta-analysis and five genetic models were merely used. While in the current meta-analysis, six genetic models were used and the publications in 2015 and 2017 were also included. Hence, our study increased the number of studies to seven individual studies in the Chinese population and offers an updated and more comprehensive result.

This study is not without limitations. Many factors can influence plasma concentrations of SUMO4, such as *SUMO4* rs237024 and rs600739 polymorphisms, diet, smoking, and hypertension (16). In addition, the current meta-analysis lacks large-scale studies on the subject. This variant has been studied only in Chinese, Japanese, and Iranian subjects. No study in other ethnicities has been found. Furthermore, no data from GWA studies on the region that includes this variant have been found. Much remains to be clarified on how the *SUMO4* gene M55V polymorphism affects patient T2DM susceptibility.

In conclusion, we found a significant association between *SUMO4* gene M55V polymorphism and T2DM risk in the current meta-analysis. Individuals with the Val allele of *SUMO4* gene M55V polymorphism may be more susceptible to T2DM in the Chinese population. This association in Japanese or Iranian population needs to be further verified in the larger samples studies. This conclusion may help researchers formulate an individual treatment strategy to prevent T2DM in the future.

## Author Contributions

Conceived and designed the meta-analysis: Y-yL and HW. Performed the meta-analysis: Y-yL, X-xY, H-yG, and GG. Analyzed the data: Y-yL. Contributed material/analysis tools: Y-yL. Wrote the manuscript: Y-yL and HK. Reference collection and data management: Y-yL and J-jW. Statistical analyses and paper writing: Y-yL, Y-hZ, and HK. Study design: Y-yL and J-jW.

## Conflict of Interest Statement

The authors declare that the research was conducted in the absence of any commercial or financial relationships that could be construed as a potential conflict of interest. The reviewer MH and handling editor declared their shared affiliation.
